# Methodologies for data collection

**DOI:** 10.1186/1753-6561-2-s3-s5

**Published:** 2008-11-14

**Authors:** Sheryl Happel Lewis, Richard Wojcik

**Affiliations:** 1 The Johns Hopkins University Applied Physics Laboratory, Laurel, MD, USA

## Abstract

**Background:**

Electronic disease surveillance systems can be extremely valuable tools; however, a critical step in system implementation is collecting data. Without accurate and complete data, statistical anomalies that are detected hold little meaning. Many people who have established successful surveillance systems acknowledge the initial data collection process to be one of the most challenging aspects of system implementation.

**Methods:**

This discussion will describe the various methods for collecting data as well as describe some of the more common data feeds used in surveillance systems today. Given that every city/region/country looking to establish a surveillance capability has varying degrees of automated data, alternative data collection methods must be considered.

**Results:**

While it would be ideal to collect automated electronic data in a real-time fashion without human intervention, data may also be effectively collected via telephone (both mobile and land lines), fax, and email. Another consideration is what type of data will be used in a surveillance system. If one data source is of high value to one locality, it should not be assumed that it will be as useful in another area. Determining what data sources work best for a particular area is a critical step in system implementation.

**Conclusion:**

Regardless of data type and how they are collected, surveillance systems can be successful if the implementers and end users understand the limitations of both the data and the collection methodology and incorporate that knowledge into their interpretation procedures.

## Background

Data are the cornerstone of any electronic disease surveillance system. For the purposes of this discussion, data are defined as any information that would be of value in a disease surveillance system.

**Figure 1:**
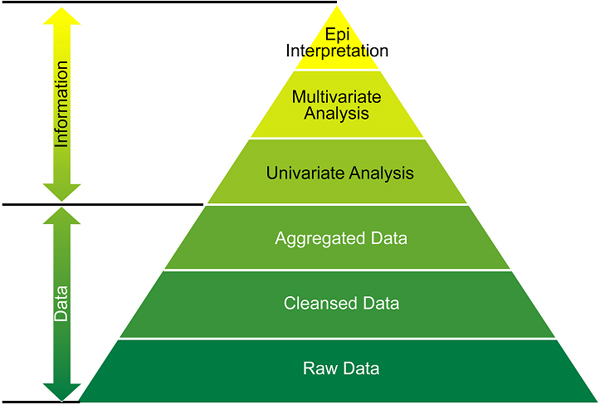
Pyramid of data formats.

Data comes in a variety of formats, from raw text line listings of patient encounters up through information gathered via analysis or end user interpretation (Figure 1) [1]. Privacy regulations in a given situation may determine the level of data that are to be shared within or between systems. For example, in a local health department in the United States, data may be collected at the individual hospital level, transmitted to the health department and then reviewed on a "per patient" basis for the purposes of assessing the health of the community. However, since the regional or state health department may not have the same perspective on the particular nuances of a community, they might only have a need to review aggregated counts or an assessment/interpretation of the data. Similarly, at the national level, again, lacking the local knowledge of recent events, they may only benefit from the analytical results or epidemiologist's interpretations of the data.

There is a distinction that can and should be made between indicator surveillance and event-based surveillance. Indicator surveillance, which is the basis for this paper, refers more to syndromic surveillance such as Electronic Surveillance System for Early Notification of Community-based Epidemics (ESSENCE), while event-based surveillance, such as the World Health Organization Early Warning and Response Network (WHO EWARN) relies more on the capture of information about events that pose a potential health risk to a particular population. Data for event-based systems can be both formal and informal with examples of formal being routine reporting systems and informal being news media and rumors [2].

Additionally, not all data sources are equal for surveillance purposes. One must be very careful to evaluate each data source thoroughly before deciding to include it in an electronic surveillance system. For example, if the data are collected and transmitted in a timely fashion but really provide little value to the end user in assessing the health of the community, it may be better not to include that data source, so as not to deplete precious resources for data acquisition, analysis, and interpretation. And although a data source can be used for a system in one locale does not mean that it can be used in a system in another locale.

## Methods

### Data source consideration

When assessing the value of data sources, the following considerations should be made:

• Availability – Consider if data are already being collected for another purpose. If so, will the system developers be able to access it?

• Privacy regulations – Research any privacy laws that may be applicable in the area of interest.

• Early indicator – Consider if a particular source has the potential to provide early notification of a problem if early detection is a system requirement.

• Coverage – Determine what population is covered by a particular data source.

• Timeliness – Understand the frequency with which data are collected and transmitted.

• Digitalization – Establish how the data are being collected and transmitted.

• Automaticity – Verify if the data are sent to a server automatically or if cued by a person.

• Reliability – Ascertain how many times the data source "drops" or is unavailable over the course of several months.

• Centralization – Determine if the data are being collected from one central point or from multiple sources.

• Cost – Understand the start-up or recurring costs associated with the data.

### Characteristics of a good data source

What makes a good data source for one type of system may not hold true for other similar systems operating in different environments. For example, if the community under surveillance is a rural area with limited access to large chain stores, spending large amounts of time and money to acquire over-the-counter pharmaceutical data will be difficult. Unlike an urban area, which might be able to acquire data from chain stores that have multiple outlets in an area, rural areas will have independently owned and operated stores that likely do not have an automated inventory system. Therefore, the time and effort needed to acquire the data will likely outweigh the benefit of the data since it is limited in its scope.

### Identifying potential data sources

When contemplating the acquisition and addition of particular data for inclusion in an electronic disease surveillance system, the developers and implementers should consider the WHO, WHAT, WHEN and HOW of each data source.

#### Who

The *WHO *of an electronic disease surveillance system will vary depending on the requirements of each system. For example, if the system is intended to pick up diseases in remote parts of a country where people have limited access to hospitals, more effort should be placed on remote data collection utilizing a laptop, personal digital assistant (PDA), integrated voice response (IVR), etc. Many of the considerations under this particular category pertain to demographic coverage.

• Will the data source cover human health, human behavior, animal health, plant health, etc.?

• Will the data source include urban or rural populations?

• What age groups will make up the majority of the data?

• What are the primary occupations of the individuals covered by this data source?

• Is the covered population subject to privacy regulations?

#### What

It is critical to assess what is trying to be gained from a particular data source and how the data will provide that information for the community in question. Questions to consider include:

• Will the data source provide traditional or non-traditional indictors?

• Is the data source an early indicator of disease?

• Is the data already being collected for another purpose?

#### When

The frequency of data transmission will vary between data sources and systems. Similarly, the frequency needs will vary by system. While one system may require that data be collected and transmitted in real-time, other systems may only need data on a weekly basis. Again, the implementers and developers need to set forth the requirements early in the system development process and clearly identify their surveillance goals.

#### How

In many locales data can be transmitted electronically via a computer; however, it is likely that in more remote environments data collection may be done via a telephone, fax machine, or the like. There is a great amount of work/research going on in this field and it is likely that in the near future more robust options for remote data collection will likely be available.

### Data in use today

There are currently many data sources that are being utilized by electronic disease surveillance systems in both countries with a high level of internet connectivity as well as those settings with very limited or no internet connectivity. In countries with a high level of internet connectivity, the focus is on the use of pre-diagnostic data for the purposes of early detection of a disease outbreak. To aid in this detection, data sources frequently used include physician office visit data, ambulance 911 calls (a.k.a. EMS), hospital emergency department visits, hospital admissions, school absentee data, pharmacy sales, nurse-hotline data, and laboratory test requests [3]. Table 1 provides examples of some data sources commonly considered to be traditional and non-traditional [4]. Important properties of these pre-diagnostic data include sensitivity, specificity, latency, and completeness.

**Table 1:** Traditional and non-traditional data sources.

**Traditional Sources**	**Non-Traditional Sources**
Emergency Department Visits	Over-the-Counter Medicine Sales
Laboratory Results (i.e., radiology, microbiology)	Poison Control Center Calls
Physician Office Visits	Water Quality Data
	Environmental Sensor Data
	School Nurse Visits
	School Absenteeism
	911 Calls/Nurse Hotline
	Media Reports

### Achieving sustainable data collection

One of the most challenging aspects of electronic surveillance systems is sustaining data acquisition. While much time and energy goes into the physical data acquisition during the implementation stages, it is equally important to consider issues surrounding sustainability in the long-term. This is a consideration that must be well thought out in the planning stages as it has been the experience of many public health entities in the United States that once a data feed has been established it is more difficult to get people to dedicate time and resources into modifying either the data elements collected, the type of transmission, or the mode of transmission [4].

If data are not currently being collected at the desired level of detail or frequency, the developers need to consider implementing electronic data collection. It is important to get the data collectors in the habit of data entry early in the process because once the task of data entry is embedded in the daily routine, sustainability is more attainable.

Likewise, it is recommended that as many variables as possible be collected at the initiation of the project. It is always better to have more information than is actually needed then to try to go back and add additional pieces later. As mentioned previously, it can be challenging to have people allocate time and resources on modifying a data feed that is functioning properly.

Data transmission methods play a large role in the success of a system. Data are only reliable as the method of data transfer. If the mode of transmission is not reliable, consider building in redundancy until the preferred method is improved upon. For example, if the goal is to transmit data electronically via the internet but the internet connection is not as robust as desired, consider implementing the internet transfer but also ask for data via flash drives until the internet connectivity improves in future years.

### Additional challenges

Once data are collected, there are inherent challenges that must be addressed. While data cleansing and processing are outside the scope of this paper, issues for consideration include workdays vs. weekends, holidays, data dropouts, data timeliness, incomplete data, and regional and cultural medical seeking behavioural differences.

## Conclusion

Selecting and maintaining appropriate data sources is a challenging aspect to the planning and implementation of electronic disease surveillance systems. Similarly, obtaining the data feeds can be a time-consuming task, so sufficient evaluation and planning must be made upfront to reduce wasting precious resources.

Data sources are not valuable unless they are complete, timely, and cover the desired population. Data does not need to be automated in order to provide high value to a system as long as the timeliness of receiving the data meets the surveillance goals. Although it would be ideal to always receive the data as quick as possible, the surveillance activity cannot place unrealistic requirements on data transmission – accept the best that a data provider can offer and aim to improve it in the future.

In summary/conclusion, careful data source evaluation and data collection planning will save time in both system implementation and day-to-day system monitoring.

## Competing interests

The authors declare that they have no competing interests.

## Authors' contributions

Both authors contributed equally to this paper.
